# Recurrence complicated with peritoneal dissemination after single-port gasless myomectomy for cellular uterine leiomyoma: A case report and literature review

**DOI:** 10.1097/MD.0000000000037444

**Published:** 2024-03-15

**Authors:** Yuanyuan Hu, Tongfu Feng

**Affiliations:** a Department of Graduate School, Hubei University of Medicine, Shiyan, China; b Department of Gynecology, Maternal and Child Health Hospital of Hubei Province, Wuhan, China.

**Keywords:** cellular uterine leiomyomas, peritoneal dissemination leiomyomatosis, recurrence, single-port gasless laparoscopic myomectomy

## Abstract

**Rationale:**

Cellular uterine leiomyomas (CL) represent the prevailing subtype among uterine leiomyomas. In this study, we report a case of recurrent peritoneal disseminated uterine fibroids 2 years after single-port laparoscopic gasless myomectomy. This article endeavors to examine the potential limitations of the aforementioned surgical procedure and outline the distinguishing features of recurrent cases with primary postoperative pathology as CL. Additionally, it aims to provide a summary of previous retrospective studies on CL and propose the existence of immunohistochemical molecules that may serve as predictors for the postoperative recurrence of cellular uterine fibroids. The ultimate objective is to enhance clinicians’ comprehension of the disease.

**Patient concerns:**

Two years ago, the patient underwent a single-port gasless laparoscopic myomectomy for uterine fibroids. Gynecological color Doppler ultrasound conducted 3 months ago revealed recurrence of uterine fibroids, and the patient experienced abdominal distension, mild urinary frequency, and constipation for the past month.

**Diagnoses:**

After the second surgical procedure, a comprehensive pathological examination and immunohistochemical analysis of both the uterine mass and metastatic lesions revealed that the definitive diagnosis was CLs.

**Interventions:**

The patient underwent the total hysterectomy, bilateral salpingectomy, pelvic adhesiolysis, omental mass resection, mesenteric mass resection, and pelvic peritoneal mass resection. All specimens were sent for rapid frozen examination and showed to be leiomyomas.

**Outcomes:**

The patient was discharged from the hospital on the 10th day after the operation. At the date of writing the article, the patient had no recurrence for 1 year and 5 months.

**Lessons:**

The single-port gasless approach did not achieve the desired reduction in fibroid recurrence, as anticipated by the surgeon. The act of pulling the tumor towards the abdominal incision for resection, on the contrary, may serve as an iatrogenic factor contributing to postoperative recurrence of CL into peritoneal dissemination leiomyomatosis. The single-port gasless assisted bag may be a more suitable option for myomectomy. The utmost effort should be made to prevent the potential recurrence of myoma caused by iatrogenic factors.

## 1. Introduction

Cellular uterine leiomyoma (CL) is a special type of uterine leiomyoma without specific symptoms. The most common clinical symptom is abnormal menstruation. The second is an asymptomatic examination. The pathological features are rich and closely arranged cells. The size and shape of the cells are relatively single, the nucleus is dense, and only individual cells appear to have atypia. The treatment of CL is the same as that of benign uterine fibroids, which is mainly based on surgery. Surgery includes myomectomy, total hysterectomy, and double adnexectomy. However, because of its borderline, easy recurrence, and malignant transformation, long-term monitoring is required after surgery. The patient had an asymptomatic physical examination as the first symptom and underwent a laparoscopic single-hole gasless myomectomy. Peritoneal disseminated uterine fibroids recurred 2 years after surgery. The case is now reported as follows:

## 2. Case presentation

**Figure 1.**
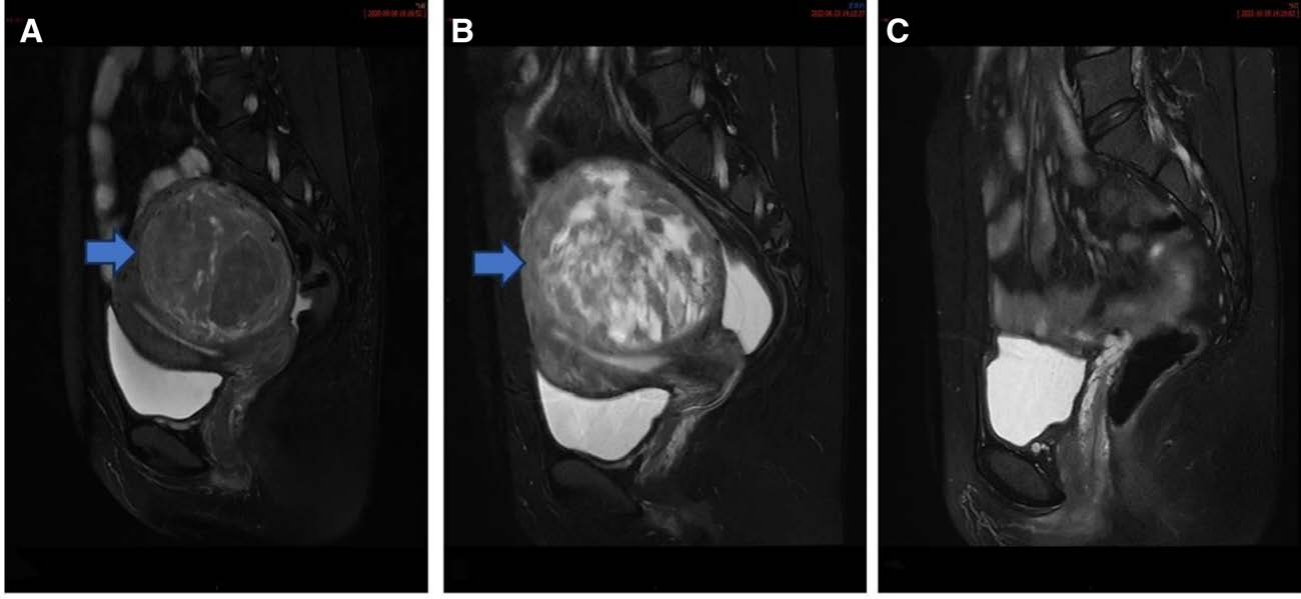
(A) A plain scan and enhancement of uterine appendages showed a 10 * 10 * 9 cm mass between the muscle walls of the posterior wall of the uterus on September 8, 2020. (B) A plain scan and enhancement of uterine appendages showed an 8.6 * 9.4 * 8.6 cm mass between the muscle walls of the posterior wall of the uterus on June 23, 2022. (C) Postoperative status of the second laparoscopic surgery.

A 38-year-old woman (G1P1A0) was admitted to the outpatient department of our hospital on June 22, 2022. Two years ago, the patient underwent single-hole gasless laparoscopic myomectomy and umbilicus plastic surgery in our hospital due to a 10 * 10 * 9 cm uterine myoma between the posterior wall of the uterus (Fig. 1A). The gasless single-hole approach platform was placed during the operation. The most protruding part of the surface of the posterior wall of the uterus was cut by a unipolar incision, the tumor was exposed, and the tumor was pulled to the abdominal incision. Postoperative pathological examination showed CL. Immunohistochemical results: CD10 (+), SMA (+), Desmin (+), and CyclinD1 (−). P53 (−), h-caldesmon (+), Ki-67 (LI about 10%). Three months ago, the gynecological color Doppler ultrasound in our hospital showed that there was a low echo of 7.0 * 6.1 * 6.0 cm in the posterior wall of the uterus. One month ago, the patient began to feel lower abdominal distension, mild frequent urination and constipation. The current physical examination prompt: gynecological examination: uterus, anterior, enlarged as 3 months of pregnancy, the rest without exception. The plain scan and enhanced MRI of the uterus and bilateral appendages showed an 8.6 * 9.4 * 8.6 cm mass between the muscle walls of the posterior wall of the uterus, sacral cyst, and pelvic effusion (Fig. 1B). Biochemical analysis, tumor markers, thinprep cytologic test + human papillomavirus, and endometrial pathological examination were negative.

**Figure 2.**
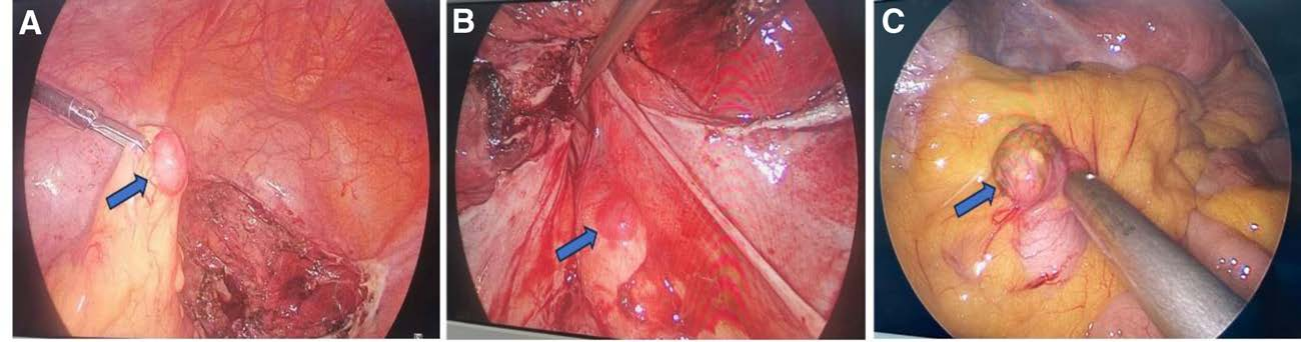
The second laparoscopic surgery showed: (A) a mass with a diameter of 2 cm was seen on the surface of the greater omentum. (B) A mass of 1.5 cm in diameter was seen in the medial peritoneum of the right uterosacral ligament. (C) A mass with a diameter of 2 cm was found on the surface of the mesentery of the sigmoid colon.

In laparoscopic surgery, we saw that the uterus was spherically enlarged, and the mass between the posterior wall muscle walls was about 8 * 8 * 7 cm. A nodule with a diameter of 1.5 cm was seen in the peritoneum of the right uterosacral ligament. The greater omentum adhered to the left abdominal wall, and a nodule with a diameter of 2 cm was seen on the surface. A mass with a diameter of 2 cm was seen on the surface of the sigmoid mesocolon, and 2 gray-white fibroid nodules were seen on the abdominal wall, with a diameter of about 1 to 2 cm (Fig. 2). The patient underwent the total hysterectomy, bilateral salpingectomy, pelvic adhesion lysis, omental tumor resection, mesenteric tumor resection, and pelvic peritoneal tumor resection.

Postoperative pathological examination showed: 10 * 9 cm CLs between the myometrium; immunohistochemical results: CD10 (+), CD225 (−), Desmin (+), SMA (+), h-caldesmon (−), ER (+), PR (+), WT1 (−), CyclinD1 (−), P53 (wild-type expression), FH (+), Ki-67 (LI: hot spot area 10%); jAZF1 (−), abdominal wall mass, greater omentum mass, mesocolon mass, pelvic peritoneal surface mass were CLs. Their immunohistochemical results: CD10 (+), CD225 (−), Desmin (+), SMA (+), h-caldesmon (+), FH (+), Ki67 (LI about 5%). The patient was discharged from the hospital on the 10th day after the operation. Due to the wild-type expression of P53, the patient was asked to follow up closely after discharge. At the date of writing the article, the patient had no recurrence for 1 year and 5 months.

## 3. Discussion

CL is the most common type of uterine fibroids, with an incidence of 17.9%. Its clinical manifestations are abnormal menstruation, abdominal pain or abdominal distension, and pelvic compression symptoms. It is easy to relapse and metastasize after an operation. The average recurrence time is 28.6 months. It belongs to the category of borderline uterine tumors and has malignant potential.^[1–3]^ Although in recent years, studies have pointed out that multi-parameter magnetic resonance imaging^[4]^ and different contrast-enhanced ultrasound^[5]^ play a certain role in the preoperative diagnosis of cellular uterine fibroids and uterine sarcoma, the current identification of the 2 mainly depends on histopathological examination and immunohistochemical markers.

At present, there is no clinical guideline for cellular uterine fibroids. Based on the existing clinical guidelines for uterine fibroids, cellular uterine fibroids belong to benign uterine lesions. The treatment is mainly based on surgery. For women with fertility needs, myomectomy is feasible. Patients without fertility needs are more recommended for total hysterectomy. The patient’s initial admission indicated a desire to preserve the uterus, thus prompting the performance of myomectomy. The introduction of minimally invasive surgery has significantly improved pain management, postoperative recovery, and scar aesthetics in the treatment of symptomatic uterine fibroids. To address the issue of specimen extraction, rotary cutter have also been developed; however, they have introduced several complications such as iatrogenic injury, endometriosis, and peritoneal dissemination leiomyomatosis.^[6]^ To mitigate the risk of peritoneal dissemination leiomyomatosis (DPL) caused by the rotary cutter and pneumoperitoneum, while also considering postoperative wound recovery and appearance, the surgeon opted for the gasless single-port approach platform during the procedure. The most convex surface of the posterior wall of the uterus was unipolarly cut to expose the tumor. The tumor was pulled to the abdominal incision and completely stripped. However, combined with the metastatic lesions observed in the abdominal wall, omentum, and sigmoid mesocolon during the second surgery, it is implied that this gasless surgical technique may act as an iatrogenic factor leading to postoperative recurrence of CL. Although no relevant studies have demonstrated a direct causal relationship between this surgical modality and the recurrence of CL into DPL, our case serves as a reminder that this surgical approach may potentially contribute to the development of peritoneal disseminated leiomyomatosis. At present, in-bag morcellation is more recommended to reduce fibroid recurrence.^[7]^ The utilization of single-port gasless assisted bag fragmentation for uterine fibroids appears to be a more favorable option in reducing the recurrence of fibroids after surgery. Therefore, the prevention of potential recurrence of iatrogenic-induced uterine fibroids should be prioritized in clinical practice.

Here we list the case reports of postoperative recurrence of patients with a clear pathological type of cellular uterine fibroids after the first operation, a total of 8 cases (Table 1).^[8–13]^ We found that the age of the patients ranged from 30 to 72 years old. The intramural fibroids, the size of the fibroids, and the number of fibroids may be high-risk factors for postoperative recurrence of CL. The recurrence sites are pelvic peritoneum, lung, mediastinum, ribs, vertebrae, fallopian tubes, abdominal wall, etc. Interestingly, when the lung is involved, the postoperative pathological examination is for leiomyosarcoma. Moreover, whether it is total hysterectomy plus double adnexectomy or myomectomy, there is a recurrence of CL after the operation. Reviewing previous case studies on CL, we found that CL has no specific symptoms.^[14]^ Compared with uterine fibroids, CL fibroids are larger in diameter and fewer in number and are not associated with endometriosis or adenomyosis,^[15]^ but this is not absolute because Umar Riaz reported a patient with solitary cecal endometriosis and extrauterine retroperitoneal cell-rich leiomyoma.^[16]^ Studies have shown that age, intramural fibroids, the number of fibroids, and the size of fibroids are independent factors for recurrence after CL.^[17]^ In our case, the patient was 38 years old, and the intramural fibroids is a high risk factor, which confirmed the possibility of recurrence in a short time after surgery.

**Table 1:** Summary of case reports of postoperative recurrence in patients with a clear pathological type of cellular uterine fibroids after the first operation.

Author and year	Age	Surgical approach	Location of myoma	Number and size of myomas	Interval recurrence time	Recurrence location	Second postoperative pathology	Prognosis
Jose et al (1999)^[8]^	72	Total uterus + double adnexectomy	Intramural	2, 15 * 12 * 10; 7 * 6 * 9	4 years	Lung	Low-grade leiomyosarcoma	No recurrence was found in 2 years follow-up.
Min-Woong Kang et al (2011)^[9]^	30	Hys-teromyomectomy	Unknown	13*12*7	3 years	Right sixth rib; sixth thoracic vertebra	Cellular uterine leiomyoma	No recurrence was found in 3 years follow-up.
Guraslan et al (2015)^[10]^	62	Total uterus + double adnexectomy	intramural	3, unknown size	10 years	Cavitas pelvica	Cellular uterine leiomyoma	Long-term follow-up
Xu Xian et al (2022)^[11]^	47	Laparoscopic hysteromyomectomy	Unknown	Unknown	6 years	Ileum, rectum, bladder, umbilicus	Leiomyoma, local active growth showed cellular leiomyoma change	Unknown
Xu Xian et al (2022)^[12]^	48	Laparoscopic hysteromyomectomy	Posterior wall of uterus	Diameter 10cm	10 years;7 months (two recurrences)	Sigmoid mesocolon, greater omentum, rectum	Leiomyoma	No recurrence was found after 9 months of follow-up.
Xu Xian et al (2022)^[11]^	48	Laparoscopic hysteromyomectomy	Unknown	Unknown	7 years	Ascending colon, right fallopian tube, right abdominal wall, right pelvic funnel ligament, right subcutaneous soft tissue.	Leiomyoma	Unknown
Xu Xian et al (2022)^[11]^	33	Laparoscopic hysteromyomectomy	Unknown	Unknown	4 years	Greater omentum, mesentery	Leiomyoma	Unknown
Terhi Ahvenainen et al (2022)^[13]^	63	Hysterectomy	Unknown	Unknown	7 years later; 9 years later (2 recurrences)	Right lung; mediastinum	Leiomyosarcoma	No recurrence within 5 years
Current case	38	Laparoscopic hysteromyomectomy	Intramural	10 * 10 * 9	2 years	Uterus, abdominal wall, greater omentum, mesocolon, pelvic peritoneum	Cellular uterine leiomyoma	No recurrence was found in 1 year and 5 months.

At present, the immunohistochemical molecular studies on uterine fibroids mainly focus on the differential diagnosis of endometrial stromal sarcoma and other benign tumors. These studies show that JAZF,^[18,19]^ MCM2,^[20]^ P16, Ki-67, P53, WT1,^[21]^ ER, and PR^[22]^ can be used for the correct diagnosis of unclear uterine smooth muscle tumors. On the prediction of the recurrence of uterine fibroids, Mills et al proposed that cell cycle markers cannot predict the recurrence of atypical uterine fibroids.^[23]^ Guerra et al found that FH defects are associated with epithelial-to-mesenchymal cell transformation, which causes tumor cell metastasis.^[24]^ Yan et al found that age, family tumor history, atypical nuclear distribution range, and mitotic count were significantly correlated with the postoperative recurrence rate of patients with FH-deficient uterine leiomyoma.^[25]^ However, whether there are immunohistochemical molecules that can predict the postoperative recurrence rate of CL to guide clinical follow-up, the probability of postoperative recurrence of FH mutation-positive and defective CL, and so on. These issues are worthy of further study.

## 4. Conclusion

CL is a prominent subtype of uterine leiomyoma, and postoperative recurrence can occur following both myomectomy and total hysterectomy procedures. The method of directly pulling the tumor into the abdominal wall incision without pneumoperitoneo through a single hole may be the iatrogenic factor of CL recurrence into DPL. The utilization of single-port gasless assisted bag fragmentation for uterine fibroids appears to be a more favorable option in reducing the recurrence of fibroids after surgery. Therefore, the prevention of potential recurrence of iatrogenic-induced uterine fibroids should be prioritized in clinical practice. Furthermore, investigating whether there are immunohistochemical markers capable of predicting the likelihood of postoperative recurrence in CL is an area worthy of further research.

## Acknowledgements

We thank the patient for participating in this case report.

## Author contributions

**Conceptualization:** Yuanyuan Hu.

**Data curation:** Yuanyuan Hu, Tongfu Feng.

**Writing – original draft:** Yuanyuan Hu.

**Writing – review & editing:** Tongfu Feng.
